# Genome-Wide Association Studies for Growth and Meat Production Traits in Sheep

**DOI:** 10.1371/journal.pone.0066569

**Published:** 2013-06-25

**Authors:** Li Zhang, Jiasen Liu, Fuping Zhao, Hangxing Ren, Lingyang Xu, Jian Lu, Shifang Zhang, Xiaoning Zhang, Caihong Wei, Guobin Lu, Youmin Zheng, Lixin Du

**Affiliations:** 1 Animal Genetics and Breeding Department, Institute of Animal Science, Chinese Academy of Agricultural Sciences, Beijing, China; 2 Animal Genetics and Breeding Department, Chongqing Academy of Animal Sciences, Chongqing, China; 3 General Office, National Animal Husbandry Service, Beijing, China; Wageningen UR Livestock Research, The Netherlands

## Abstract

**Background:**

Growth and meat production traits are significant economic traits in sheep. The aim of the study is to identify candidate genes affecting growth and meat production traits at genome level with high throughput single nucleotide polymorphisms (SNP) genotyping technologies.

**Methodology and Results:**

Using Illumina OvineSNP50 BeadChip, we performed a GWA study in 329 purebred sheep for 11 growth and meat production traits (birth weight, weaning weight, 6-month weight, eye muscle area, fat thickness, pre-weaning gain, post-weaning gain, daily weight gain, height at withers, chest girth, and shin circumference). After quality control, 319 sheep and 48,198 SNPs were analyzed by TASSEL program in a mixed linear model (MLM). 36 significant SNPs were identified for 7 traits, and 10 of them reached genome-wise significance level for post-weaning gain. Gene annotation was implemented with the latest sheep genome *Ovis_aries*_*v*3.1 (released October 2012). More than one-third SNPs (14 out of 36) were located within ovine genes, others were located close to ovine genes (878bp-398,165bp apart). The strongest new finding is 5 genes were thought to be the most crucial candidate genes associated with post-weaning gain: s58995.1 was located within the ovine genes *MEF2B* and *RFXANK*, OAR3_84073899.1, OAR3_115712045.1 and OAR9_91721507.1 were located within *CAMKMT*, *TRHDE*, and *RIPK2* respectively. *GRM1*, *POL*, *MBD5*, *UBR2*, *RPL7* and *SMC2* were thought to be the important candidate genes affecting post-weaning gain too. Additionally, 25 genes at chromosome-wise significance level were also forecasted to be the promising genes that influencing sheep growth and meat production traits.

**Conclusions:**

The results will contribute to the similar studies and facilitate the potential utilization of genes involved in growth and meat production traits in sheep in future.

## Introduction

With the increasing popularity of meat production in sheep industry, geneticists and breeders pay more attention to sheep growth and meat production traits. In the past decades, a mass of quantitative trait loci (QTL) were found by candidate gene approach and genome scanning technology in livestock. QTL plays an essential role in the genetic evaluation of breeding animals. Numerous QTL studies for different quantitative traits have been performed in pig, cattle, chicken and sheep [1], [2], [3], [4]. Unfortunately, a small number of QTL have been identified in sheep with only 789 reported from a genome scan based on marker-QTL linkage analysis [5](updated on May 15^th^,2013). However, the QTL confidence interval is relatively long, and it is difficult to identify specific genes influencing target quantitative traits. Thus, some novel gene identifications may be overlooked. With the development of high throughput SNP genotyping technologies, genome-wide association studies (GWAS) have been widely applied to detect and localize candidate genes for quantitative traits in different species, which brings more ideas to increase the efficiency of animal breeding and selection [6], [7], [8].

In human GWAS, investigators mainly focus on the inherited basis of human biology and diseases in order to improve treatment or produce useful diagnostic or predictive tests [9], [10], [11], [12]. In animals, geneticists are more concerned with economic traits, genomic prediction, and genomic evaluation to accelerate genetic improvement [8], [13]. During the past 7 years, GWAS have identified several important candidate genes and thousands of papers were published. Nevertheless, only a small number of GWAS mentioned sheep due to limited information available in sheep genome. Most of DNA sequence in sheep is discontinuous, whether QTL studies or known genes, the number is the least. We only know about 700 genes before sheep genome *Ovis_aries_v*3.1 released in October 2012 [14], [15]. And in these articles, the results associated more diseases than production: for example, *Chondrodysplasia* (a condition in which the legs are malformed) was found to be associated with a group of consecutive SNP markers [16]. *Paratuberculosis* (*Johne*'*s disease*) caused by Mycobacterium avium subspecies paratuberculosis has also been reported in sheep GWAS [17]. The mutation of *DMP1* gene was indentified to be associated with inherited rickets of Corriedale sheep by GWAS [18].The latest sheep GWA study focused on milk production traits in dairy sheep [19]. Moreover, GWAS and fine mapping of QTL were performed on chromosome 21 focusing on body weight only [20].

Currently, knowledge of the major genes or QTL associated with sheep growth and meat production traits are comparatively limited, and few of these QTL offer useful information in production. *Myostatin* (*MSTN*) is one of the best-characterized genes known to influence the movement and growth of muscle cells. In 1998, 1999 and 2002, QTL related to muscle in sheep were identified on OAR2, which also contained *MSTN* [21], [22], [23]. Another studies showed that several QTL were found close to *MSTN* on OAR2 in sheep [4], [24], [25], [26]. *Callipyge*(*CLPG*) is another well-known major gene in sheep that has been shown to significantly enhance the lean-meat percentage [27], [28]. *Carwell* gene was found in Poll Dorset ram in Australia in the 1980 s. It was located on OAR18 and close to the gene *CLPG* [29]. Researchers guessed that they might be mutual alleles. Compared with *CLPG*, *Carwell* affects rib-eye muscles but has no effect on fat thickness and body weight [30]. QTL around *Carwell* was also found in UK Texel sheep [31].

The main objective of this study is, by using Illumina OvineSNP50 BeadChip and GWAS methodology [32], to identify those significant SNPs associated with growth and meat production traits at genome level, and to explore and forecast the major candidate genes in sheep. It is worth stressing that, we performed a GWA study in a mixed sheep population without distinguishing family and variety. The filtered SNP loci may be used as a preliminary foundation for further replication studies and eventually to determine causal mutations associated with enhanced growth and meat production traits in sheep.

## Materials and Methods

### Animal resources

The sheep population used in this study consists of 69 Sunit sheep(57 males and 12 females),161 German Mutton sheep (71 males and 90 females) and 99 Dorper sheep(49 males and 50 females). A total of 329 sheep were purebred and individuals were randomly selected from Inner Mongolia Sunit Purebred Sheep Stud (Xilin Gol League, Inner Mongolia, China), Inner Mongolia German Mutton Purebred Sheep Stud (Xilin Gol League, Inner Mongolia, China)and Tianjin Aoqun Animal Husbandry Propriety Limited(Jinghai County, Tianjin, China), respectively. There were not any family structure and half sib family in the selected sheep. The three studs are all located in the north of China, sheep were raised with standardized management, and the feeding environments were almost the same.

### Measurement of growth and meat production traits

This study mainly focused on phenotypic traits associated with sheep growth and meat production. Body weight was recorded when the lamb was born, weaned, and at 6 months old. Body sizes including height at withers, chest girth, and shin circumference were obtained at 6 months. We also estimated the fat thickness and eye muscle area by B ultrasonic (Aquila Vet, Holland) while the sheep was alive at 6 months [33]. From the above records, 11 traits were concerned finally: birth weight (BWT), weaning weight (WWT), 6-month weight (SMWT), pre-weaning gain (PRWG), post-weaning gain (PWG), daily weight gain (DWG), eye muscle area (EMA), fat thickness (FT), height at withers (HW), chest girth (CG), and shin circumference (SC). All animal experiments were permitted in the farms in compliance with the Law of Animal Husbandry in People's Republic of China(Dec 29,2005).

As a side note, 6 months mentioned in this study refers to 190 days. To make convenience of computation, we have adjusted the raw data by conventional linear statistics. Weaning weight is the adjusted weight when sheep was 125 days.

### Sample and genotyping

Blood samples were collected using traditional method. Genomic DNA was extracted from blood samples using the TIANamp Blood DNA Kit (Tiangen Biotech Company Limited,Beijing,China). DNA was quantified and genotyped using the Illumina OvineSNP50 BeadChip containing 54,241 SNPs.

The genotyping platform used in this research was Infinium II Multi-sample Assay. SNP chips were scanned using iScan and analyzed using Illumina GenomeStudio (Illumina, Inc.9885 Towne Centre Drive, San Diego, CA 92121 USA). To assess the technical reliability of the genotyping panel, two or more randomly selected DNA samples were genotyped and over 99.9% identity was obtained. The SNP array data discussed in this study have been deposited in Gene Expression Omnibus (GEO) of National Center for Biotechnology Information and are accessible through GEO Series accession number GSE46231.

### Genotype quality control

We used the PLINK software(v1.07, http://pngu.mgh.harvard.edu/~purcell/plink) to exclude individuals and remove SNPs from the 329 individuals and 54,241 SNPs. An individual was excluded if (1) more than 10% of the genotype was missing, (2) an error occurred in sex testing, or (3) it was a duplicate sample. A SNP was removed if (1) its call rate was less than 90%, or (2) its minor allele frequency (MAF) was less than 3%. However, we did not remove SNPs based on the Hardy-Weinberg Equilibrium (HWE) *P-* value. When we considered the three sheep varieties as a whole population for analysis, the population was not in agreement with HWE, and positive sites would have been missed. Following such quality control steps, 319 individuals and 48,198 SNPs were used for the subsequent GWAS analysis.

### Genome-wide association analysis

GWAS was performed using TASSEL 3.0 (http://www.maizegenetics.net) based on a mixed liner model (MLM),
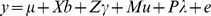
where *y* is the vector of the phenotypic values of interest, is the population mean, *b* is a vector containing fixed effects including breed and sex, is a vector of the SNP effect, *u* is a vector of the polygenic effect,is a vector of birth weight. *X* and *Z* are the known design matrices for the fixed effects *b* and ,respectively. *M* is the known matrices for the random effect *u*. *P* is the matrix for birth weight. is the unobserved vector of random residuals.

Here we assumed that *u* and follow normal distribution. *u*∼*N*(0,), where  = K. is an unknown additive genetic variance and K is the kinship matrix. *e*∼*N* (0, R), R = I, is the unknown residual variance.Though no pedigree is available in this population, we considered the relationship between any two individuals. TASSEL software provides a function to estimate kinship matrix from a set of random markers covering the whole genome. We adopted it to correct possible relationship in the population.

Moreover, linear regression analysis suggested that birth weight had high correlation with the other meat production traits. So we added birth weight values as a continuous covariant in the model when we calculated the phenotypic values of the other traits except birth weight.

Principle component analysis (PCA) was initially used in the model. Ten main principle components (PCs) were evaluated using 17,082 independent SNP markers to correct for population structure estimated using the v3.0 EIGENSOFT software [34], [35].These SNPs were pruned using the indep-pairwise option in PLINK, with a window size of 1500 SNPs and r^2^ threshold of 0.2. In this study, all samples have clear resources and the population is from three separate breeds. Therefore, we ultimately considered the breed effect instead of PCs.

### Statistical inference

During the analysis, the Bonferroni method was used to adjust for the multiple SNP loci detected. We concluded that a SNP was significant at the genome-wise significance level with a raw *P*-value of <0.05/*N*, where *N* is the number of SNP loci tested in this study. Likewise, chromosome-wise level refers to the raw *P*-value <0.05/n, where n is the number of SNP loci on each chromosome tested in this study.

### Population stratification assessment

Confounding due to population stratification is a major issue in GWAS [36]. In this study, although the resources of the three breeds were very clear, we still examined the distribution of the test statistics obtained from the numerous association tests. We also assessed their deviation from the expected distribution of no SNPs being associated with the trait of interest using a quantile-quantile (Q-Q) plot, which is commonly used to analyze population stratification in GWAS.

### Gene annotation

We used well-known websites such as UCSC Genome Bioinformatics [37], National Center for Biotechnology Information (NCBI)[38], especially, the latest sheep genome *Ovis_aries*_*v*3.1[39] to identify relationships between significant SNPs and ovine genes. Due to the structural imperfection and laggard research on sheep genome(before October,2012), we also referenced to the genomic information of other species such as human, bovine, mouse, and rat to predict correlations between SNPs and their genes.

## Results

### Phenotype statistics and SNP distribution before and after quality control

Descriptive statistics of the phenotypic observations of the 11 growth and meat production traits are presented in Table **1**. After quality control, 10 individuals were excluded, leaving 319 sheep for the association analysis. Additionally, we removed 3,758 SNPs with call rates less than 90% and 5,626 SNPs with MAF less than 0.03. A total of 48,198 SNPs passed these quality-control filters and were retained in the dataset.

**Table 1:** Descriptive statistics of 11 sheep growth and meat production traits.

Traits	Mean	Standard deviation	Minimum	Maximum	Standard error
birth weight	3.89	0.90	1.70	6.80	0.0508
weaning weight	28.98	6.67	13.75	51.84	0.3742
6-month weight	39.11	7.75	18.71	58.92	0.4348
eye muscle area	9.51	3.11	2.62	19.24	0.1746
fat thickness	0.50	0.13	0.26	0.86	0.007
pre-weaning gain	0.20	0.05	0.09	0.37	0.0030
post-weaning gain	0.16	0.08	−0.03	0.52	0.0043
daily weight gain	0.20	0.04	0.08	0.28	0.0022
height at withers	61.126	3.09	51.2	69.0	0.1734
chest girth	86.426	8.28	62.4	104.7	0.4642
shin circumference	8.592	1.10	5.7	10.6	0.0615

The distributions of the remaining SNPs before and after filtering and the average distances between adjacent SNPs on each chromosome are given in Table **2**. After quality control, the number of SNPs on each chromosome varied from 661 on OAR24 to 5,192 on OAR1, and the adjacent distance ranged from 48.91 kb on OAR8 to 63.44 kb on OAR24.

**Table 2:** Distributions of SNPs before and after quality control and the average distances between adjacent SNPs on each chromosome.

Chromosome	No. SNPs		Length of Chromosome(bp)	Average distance (kb)	
	Before QC	After QC		Before QC	After QC^a^
1	5894	5192	275612895	46.76	53.05
2	5472	4926	248993846	45.50	50.48
3	4972	4432	224283230	45.10	50.55
4	2686	2398	119255633	44.40	49.63
5	2346	2101	107901688	46.00	50.82
6	2580	2299	117031472	45.36	50.73
7	2257	1999	100079507	44.34	49.79
8	2058	1852	90695168	44.07	48.91
9	2133	1889	94726778	44.41	49.84
10	1840	1617	86447213	46.98	52.57
11	1175	1055	62248096	52.98	58.16
12	1709	1533	79100223	46.28	51.48
13	1692	1510	83079144	49.10	54.88
14	1173	1044	62722625	53.47	59.87
15	1683	1484	80923592	48.08	53.95
16	1576	1392	71719816	45.51	51.29
17	1423	1274	72286588	50.80	56.57
18	1413	1273	68604602	48.55	52.98
19	1245	1102	60464314	48.57	54.55
20	1139	989	51176841	44.93	50.91
21	883	786	50073674	56.71	63.35
22	1110	975	50832532	45.80	51.86
23	1132	1007	62330649	55.06	61.56
24	743	661	42034648	56.57	63.44
25	998	892	45367442	45.46	50.74
26	912	807	44077779	48.33	54.32
X	1481	1313	135437088	91.45	102.87
0^b^	516	396			

a: Derived from the latest sheep genome sequence assembly (*Ovis*_*aries*_*v*3.1) (http://www.ncbi.nlm.nih.gov/assembly/457978/)

b: These SNPs are not assigned to any chromosomes.

The original positions of these SNPs were based on *Ovis_aries*_v1.0 genome information [15]. By using the blast tool [39],we afresh positioned the probe sequence of Illumina SNP chip in latest *Ovis_aries*_*v*3.1 genome to get the new positions (Table 3), which helps us to run the gene annotation with higher reliability.

**Table 3:** Chromosome-wise significant (p<0.05) SNPs associated with growth and meat production traits.

Traits*	Genome- wise ad justed *P* value	Chr.	Chromo some-wise adjusted *P* value	SNP	Position v1.0(bp)	Position v3.1(bp)	Nearest gene		Raw *P* value
							Name	Distance#(bp)	
**PWG**	**6.13E-06**	**8**	2.35E-07	**OAR8_75441328.1**	75441328	70297581	**GRM1**	−58339	**1.27E-10**
	**9.35E-06**	**17**	2.46E-07	**OAR17_34475530.1**	34475530	31550788	**POL**	263208	**1.94E-10**
	**9.66E-06**	**5**	4.24E-07	***s58995.1***	3983329	3858663	***MEF2B, FXANK***	*within*	**2.00E-10**
	**1.00E-05**	**3**	9.28E-07	***OAR3_84073899.1***	84073899	79511180	***CAMKMT***	*within*	**2.08E-10**
	**1.02E-05**	**2**	1.04E-06	**OAR2_169649708.1**	169649708	160096561	**MBD5**	−158891	**2.11E-10**
	**1.14E-053**		1.05E-06	***OAR3_115712045.1***	115712045	108653757	***TRHDE***	***within***	**2.36E-10**
	**9.51E-05**	**20**	1.96E-06	**s72649.1**	17129083	16315464	**UBR2**	43239	**1.97E-09**
	**1.35E-02**	**9**	5.31E-04	***OAR9_91721507.1***	91721507	86514456	***RIPK2***	***within***	**2.81E-07**
	**1.91E-02**	**2**	1.95E-03	**s09135.1**	203019174	191583896	RPL7	−1801	**3.96E-07**
	**4.98E-02**	**2**	5.09E-03	**OAR2_19203817.1**	19203817	18820353	**SMC2**	49947	**1.03E-06**
		1	1.08E-02	OAR1_227587917.1	227587917	210935333	NLGN1	237278	2.07E-06
		20	3.16E-03	OAR20_1719751_X.1	1719752	1893550	EPB41L3	398165	3.17E-06
		1	2.19E-02	***OAR1_35717733.1***	35717733	34769254	***C1ORF87CYP2J***	***within***	4.20E-06
		14	5.79E-03	OAR14_35183176.1	35183176	33776121	CHMP5	−878	5.59E-06
		3	3.25E-02	***OAR3_84882715.1***	84882715	80279564	***LRPPRC***	***within***	7.28E-06
		18	1.49E-02	OAR18_36863544.1	36863544	35165454	TGIF1	−271401	1.17E-05
		21	1.18E-02	***s05205.1***	31657570	28262954	***STT3A***	***within***	1.48E-05
		5	3.94E-02	s32354.1	1757535	1624037	ADAMTS2	85810	1.86E-05
		9	3.66E-02	OAR9_64502345.1	64502345	61359181	TRPS1	170727	1.93E-05
		11	2.37E-02	***OAR11_58504281.1***	58504281	54623411	***SRP68***	***within***	2.24E-05
		14	2.66E-02	***OAR14_40762191.1***	40762191	39153072	***HYDIN***	***within***	2.57E-05
		13	4.49E-02	s16261.1	38333759	34941844	LSM3	−86989	2.96E-05
		16	4.55E-02	OAR16_61248510.1	61248510	56087987	MYO10	28730	3.26E-05
		21	3.56E-02	***OAR21_31060233.1***	31060233	27667519	***CCDC15***	***within***	4.46E-05
		11	4.89E-02	***OAR11_42487494.1***	42487494	40005361	***MSL1***	***within***	4.62E-05
		11	4.90E-02	s48574.1	29691860	28019509	NTN1	11838	4.62E-05
		22	4.61E-02	OAR22_2914532.1	2914532	2285531	ZWINT	199775	4.82E-05
PRWG		3	5.94E-03	s55067.1	231892354	213928939	PLA2G6	−1200	1.33E-06
		19	2.55E-03	***s34745.1***	54046219	51282206	***PFKFB4***	***within***	2.31E-06
DWG		8	1.55E-02	OAR8_16297646.1	16297646	14677113	TRDN	116188	8.37E-06
		26	1.58E-02	s16551.1	43292628	38239139	OXSM	261544	1.93E-05
		26	1.78E-02	s52984.1	44026341	38903633	RARB	−21112	2.18E-05
CG		9	3.56E-03	OAR9_55775007.1	55775007	53307896	LRRC2	131402	1.88E-06
		9	3.08E-02	OAR9_55809751.1	55809751	53344689	LRRC2	94609	1.63E-05
SC		25	2.99E-02	***OAR25_31570574.1***	31570574	30210216	***ADK***	***within***	3.35E-05
		24	4.36E-02	***OAR24_13169307.1***	13169307	11611963	***SHISA9***	***within***	6.62E-05
SMWT		8	3.20E-02	?OAR8_16297646.1	16297646	14677113	TRDN	116188	1.73E-05
		26	1.61E-02	?s16551.1	43292628	38239139	OXSM	261544	1.97E-05
		26	3.57E-02	?s52984.1	44026341	38903633	RARB	−21112	4.38E-05
WWT		3	7.94E-03	?s55067.1	231892354	213928939	PLA2G6	−1200	1.78E-06
		19	2.81E-03	?***s34745.1***	54046219	51282206	***PFKFB4***	***within***	2.55E-06

Genome-wise significant SNPs are labeled in bold.

SNPs located within known ovine genes are labeled in italics.

# Positive value denotes the gene located downstream of SNP, negative value denotes the gene located upstream of SNP.

* PWG: post-weaning gain; PRWG: pre-weaning gain; DWG: daily weight gain; CG: chest girth; SC: shin circumference; SMWT: 6-month weight; WWT: weaning weight.

? 6-month weight showed a linear relationship with daily weight gain. Weaning weight also showed a linear relationship with pre-weaning gain. Thus, there were five significant repeating SNPs.

### GWA analyses

**Figure 1:**
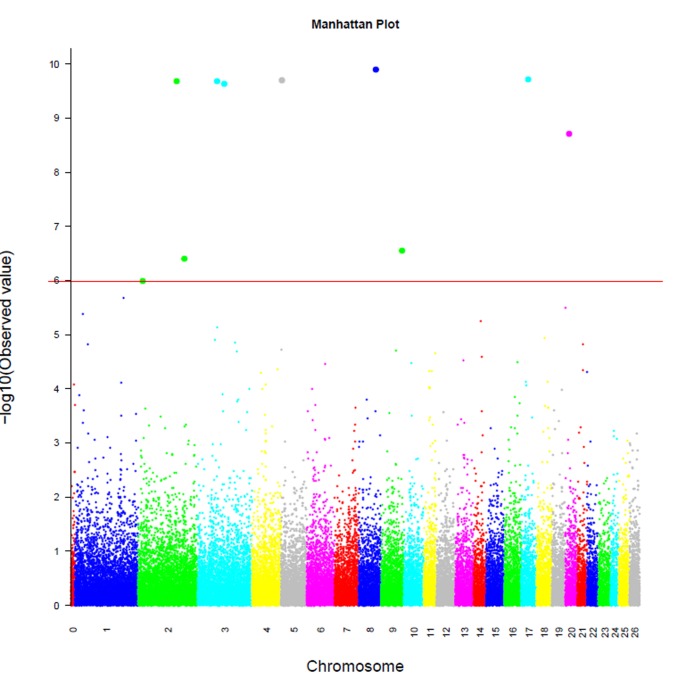
Genome-wide plot of -log_10_(*p*-values) for association of SNP loci with post-weaning gain. Chromosomes 1–26 are shown separated by color. Fig. The horizontal line indicates the genome-wise significance levels (−log_10_(1×10^−6^).

The profiles of the *P*-values (in terms of –log(*P*)) of all tested SNPs are show in Figure 1. The details of significant SNPs for all production traits are shown in Table 3, which presents a statistical signal overview of the associated SNPs across the genome. Table 3 includes the genome-wise adjusted *P* value, chromosome-wise adjusted *P* value, significant SNPs, SNP position in *Ovis_aries*_*v*1.0 and *Ovis_aries*_*v*3.1, the nearest known ovine genes, and the raw *P* value corresponding to different traits. In total, 36 significant SNPs at the chromosome-wise level were identified for PWG, PRWG, DWG, CG, SC, SMWT and WWT. Among these significant SNPs, 10 of them achieved genome-wise significance levels (indicated in bold in Table 3) with post-weaning gain only. The total numbers of distinct SNPs that were significant at the chromosome-wise and genome-wise levels were 36 and 10, respectively. No significant SNPs were identified for BWT, EMA, FT, or HW.

### Post-weaning gain (PWG)

As seen from Table 3, a total of 27 SNPs significantly associated with PWG traits were identified, 11 SNPs were found within regions of known ovine genes, and the others were located 878 bp-398,165 bp apart from the nearest known ovine genes (*CHMP5* and *EPB41L3*). A total of 10 SNPs reached genome-wise significant level, 4 of which were within the known ovine genes: *MEF2B*, *RFXANK*, *CAMKMT*,*TRHDE*, and *RIPK2*. The 11 SNPs were found not only within the genes in the sheep genome but also within the same genes in human, cattle, mouse, and rat genome (Table 4).

**Table 4:** BLAST results for the sequence between 500kb downstream and 500 kb upstream of the SNPs in sheep compared with other species (human, cattle, mouse, and rat)**.**

Traits*	SNP name	Nearest gene(human)		Nearest gene(cattle)		Nearest gene(mouse)		Nearest gene(rat)	
		Name	Distan ce (bp)	Name	Distan ce (bp)	Name	Distan ce (bp)	Name	Distan ce (bp)
**PWG**	**OAR8_75441328.1**	GRM1, GRM5 LOC440040	within	GRM1	within	Grm1 & Grm5	within	Grm1 & Grm5	within
	**OAR17_34475530.1**	SLC16A10	12688bp	SLC16A10	12533	Slc16a10	12509	Slc16a10	12688
	**s58995.1**	MEF2BNB-MEF2B	within	LOC10027185- MEF2B &MEF2B	within	Mef2b	2270	Mef2b	2270
	**OAR3_84073899.1**	SIX3, CAMKMT	within	CAMKMT	within	1700106N22Rik	within	Camkmt	within
	**OAR2_169649708.1**	MBD5	within	PTMA	27547	Mbd5	within	Ptma	27582
	**OAR3_115712045.1**	TRHDE	within	SS18L2	***30583599***	Trhde	within	Trhde	within
	**s72649.1**	UBR2	40470	UBR2	48709	Ubr2	40471	Ubr2	48709
	**OAR9_91721507.1**	RIPK2	within	RIPK2	within	Ripk2	within	Ripk2	within
	**s09135.1**	GYPC	6455	GYPC	6493	Glsp	431996	Gls	431997
	**OAR2_19203817.1**	SMC2	48797	EPC2	***149951842***	Smc2	50927	Smc2	50927
	OAR1_227587917.1	SNORA56	166711	NLGN1	243565	Nlgn1	242153	Nlgn1	243438
	OAR20_1719751_X.1	KIAA0494	320816	ALKBH8	31039	Alkbh8	31040	Alkbh8	31041
	OAR1_35717733.1	C1orf87	within	C3H1orf87	within	Gm12695	within	RGD1560146	within
	OAR14_35183176.1	DYNC1LI2	35378	DYNC1LI2	37759	Dync1li2	35379	Dync1li2	35379
	OAR3_84882715.1	LRPPRC	within	ABCG8	53517	Lrpprc	within	Lrpprc	within
	OAR18_36863544.1	NOVA1	421431	NOVA2	423505	Nova1	418477	–	–
	s05205.1	STT3A & STT3B	within	STT3A, STT3B	within	Stt3a, Stt3b	within	Stt3a, Stt3b	within
	s32354.1	ADAMTS2	78991	ADAMTS2	78991	Adamts2	78993	Adamts2	78992
	OAR9_64502345.1	TRPS1	116951	TRPS1	170106	Trps1	116986	Trps1	117635
	OAR11_58504281.1	MGRN1SRP68-intron	within	SRP68	within	Srp68	within	Srp68	within
	OAR14_40762191.1	HYDIN	within	TCEAL8	71822	Hydin	within	Ftsjd1	95835
	s16261.1	BAMBI	179619	BAMBI	178773	Bambi	177047	Bambi	180149
	OAR16_61248510.1	MYO10	28559	MYO10	28803	Myo10	85115	Myo10	28630
	OAR21_31060233.1	CCDC15	within	HEPACAM	47431	Ccdc15	within	Slc37a2	65171bp
	OAR11_42487494.1	CASC3, MSL1	within	CASC3	21338	Msl1	within	Msl1	within
	s48574.1	NTN1	11893	NTN1	11893	Ntn1	within	Ntn1	11893
	OAR22_2914532.1	PCNP	315292	ZWINT	472982	–	–	–	–
**PRWG**	s55067.1	PLA2G6	within	MAFF	25156	Pla2g6	within	Pla2g6	1181
	s34745.1	PFKFB4 & PFKFB1	within	PFKFB4 & PFKFB1	within	Pfkfb4, Pfkfb1	within	Pfkfb4, Pfkfb1	within
**DWG**	OAR8_16297646.1	TRDN	136486	EPM2A	***58578183***	Trdn	136486	Trdn	136735
	s16551.1	OXSM	268954	FGF14	171999	Oxsm	268958	Oxsm	268958
	s52984.1	RARB	35425	RARG	162202	Rarb	35693	Top2b	116254
**CG**	OAR9_55775007.1	PEX2	281921	MRPS17	80917	Pxmp3	296048	Pex2	296044
	OAR9_55809751.1	PEX2	316665	MRPS17	46173	Pxmp3	330792	Pex2	330788
**SC**	OAR25_31570574.1	ADK	within	ADK	within	Adk	within	Adk	within
	OAR24_13169307.1	SHISA9	within	SHISA9	within	Shisa9	within	Cpped1	144396
**SMWT**	?OAR8_16297646.1	TRDN	136486	EPM2A	***58578183***	Trdn	136486	Trdn	136735
	?s16551.1	OXSM	268954	FGF14	171999	Oxsm	268958	Oxsm	268958
	?s52984.1	RARB	35425	RARG	162202	Rarb	35693	Top2b	116254
**WWT**	?s55067.1	PLA2G6	within	MAFF	25156	Pla2g6	within	Pla2g6	1181
	?s34745.1	PFKFB4 & PFKFB1	within	PFKFB4 & PFKFB1	within	Pfkfb4, Pfkfb1	within	Pfkfb4, Pfkfb1	within

Genome-wise significant SNPs are labeled in bold.

* PWG: post-weaning gain; PRWG: pre-weaning gain; DWG: daily weight gain; CG: chest girth; SC: shin circumference; SMWT: 6-month weight; WWT: weaning weight.

? 6-month weight showed a linear relationship with daily weight gain. Weaning weight also showed a linear relationship with pre-weaning gain. Thus, these traits are correlated with the same genes in other species.

BLAST results for the sequence between more than 500 kb downstream and more than 500kb upstream of the SNPs in sheep compared with other species(human, cattle, mouse, and rat) are labeled in bold and italics.

The BLAST work was done before the latest version3.1 sheep genome released in October 2012.

### Pre-weaning gain (PRWG)

PRWG was linearly correlated with WWT. As a result, 2 SNPs were significantly associated with both PRWG and WWT: s34745.1 was located within the known ovine *PFKFB4* gene, and s55067.1 was located 1,200 bp upstream of the known ovine gene *PLA2G6*. Both SNPs were found within regions of known genes from four other species (Table 4).

### 6-month weight (SMWT)

SMWT showed a linear relationship with DWG. There were 3 significant SNPs associated with both SMWT and DWG. The SNPs were located 21,112 bp-261,544 bp apart from the nearest known ovine genes (*RARB* and *OXSM*).

### Chest girth (CG)

As presented in Table 3, two SNPs significantly associated with CG trait were identified and located 94,609 bp-131,402 bp apart from the same nearest known ovine genes as *LRRC2*.

### Shin circumference (SC)

As seen in Table 3, two SNPs significantly associated with SC were identified within the ovine known genes *ADK* and *SHISA9*. They were also found in the same genes in human, cattle, mouse, and rat genome (Table 4).

### Q-Q plots

**Figure 2:**
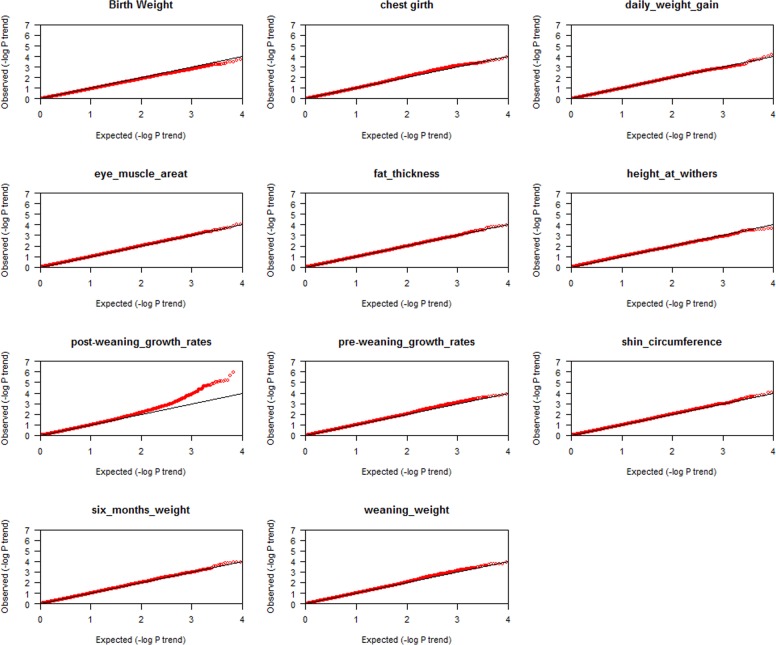
Quantile-quantile (Q-Q) plots of genome-wide association results for 11 meat production traits. Under the null hypothesis of no association at any SNP locus, the points would be expected to follow the slope lines. Deviations from the slope lines correspond to loci that deviate from the null hypotheses.

The Q-Q plots for the test statistics are shown in Figure 2. X-axis are the expected p-values under null hypothesis and on the y-axis are the observed p-values. Based on these plots, there is no population stratification in the analysis data. From the post-weaning gain Q-Q plot, the observed SNPs were greater than the expected SNPs, resulting that SNPs were associated with PWG at the adjusted genome-wise significance level.

### Population stratification

**Figure 3:**
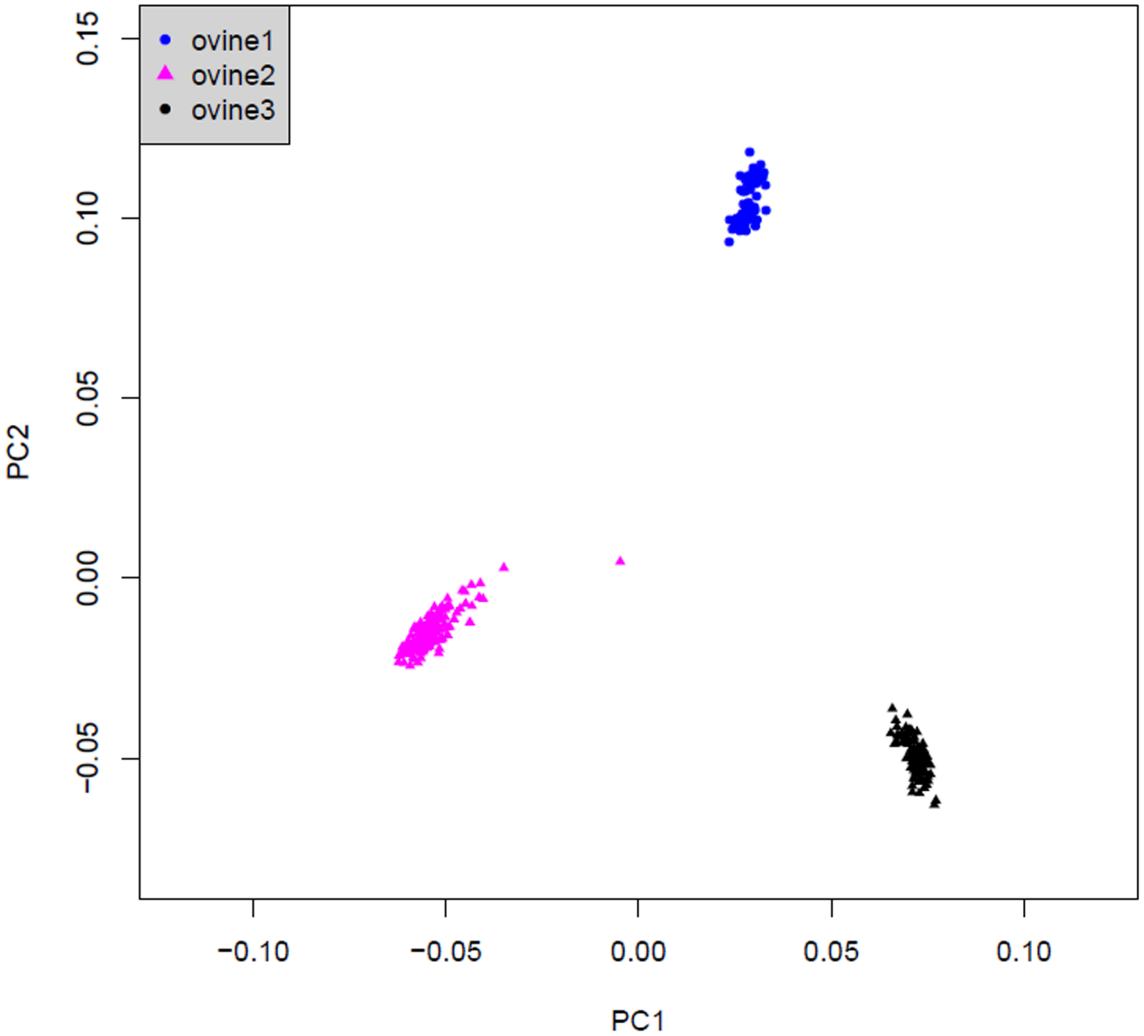
principal-component analysis for population stratification in three sheep breeds.

Principle component analysis (PCA) shows that three sheep breeds were clear and distributed separately in Figure 3, Which could be used in the model to check the population stratification.

## Discussion

In this study, we performed a GWA study for 11 growth and meat production traits using a random design in sheep population. GWA studies have been applied to many species but rarely to sheep. To our knowledge, this is one of the earliest GWA studies for so many growth and meat production traits using Illumina OvineSNP50 BeadChip. TASSEL, which has been commonly used in GWAS, was employed to analyze associations between SNPs and phenotypes [40], [41]. Gene annotation was implemented with the latest sheep genome *Ovis_aries*_*v*3.1 sequence. Therefore, analysis results can be used to identify and explore novel candidate genes, even to perform functional analysis of promising genes later.

Just before October 2012, the sheep genome information showed only 713 genes [15]. The number increased to more than 20,000 after *Ovis_aries*_v3.1 genome sequence released at the end of 2012[39]. More than 20% genes are located on OAR2 and OAR3[15]. Among the detected QTL, there are 148 on OAR2,72 on OAR1 and 67 on OAR3, which are the chromosomes containing the highest number of QTL [5](updated on May 15^th^). In our study, 36 significant SNPs were detected for 7 growth and meat production traits, and 10 of them reached genome-wise significance and were identified within or close to some ovine genes. Among the 10 SNPs, 3 were close to the genes *RPL7*(1,801bp apart), *SMC2*(49,947 bp apart), and *MBD*5(158,891 bp apart) on OAR2, and 2 of them were located within known ovine genes *CAMKMT* and *TRHDE* on OAR3. Two other SNPs reaching chromosome-wise significance were on OAR3 too, one was located within *LRPPRC* for PWG trait and the other was close to *PLA2G*6(1,200 bp apart) for both PRWG and WWT trait. There are two SNPs associated with PWG on OAR1. This is consistent with above evidence and it suggests that we could pay more attention to OAR1, OAR2 and OAR3 in sheep genome. The rest 5 significant SNPs at genome-wise significance level were within or close to genes *MEF2B*, *RFXANK*, *RIPK2*, *GRM1*, *POL*, and *UBR2*. According to the results, we gave a preliminary presumption that these genes are candidate genes for post-weaning gain, which is one of the growth and meat production traits we concerned.

*MEF2*B,myocyte enhancer factor 2B,belonging to *MEF2* gene family, has been reported to play an important role in development and differentiation of muscle cells [42], [43], [44], [45], [46]. It is involved in muscle-specific and growth-factor-related transcription and distributed widely in the tissues of fruit fly, zebra fish, mouse, and human. *MEF2B* expresses in heart and skeletal muscle in human body. Another study showed that *MEF2B* was associated with growth and meat production traits in pigs [47]. In our study, s58995.1 was identified within the intron of *MEF2*B for post-weaning gain, which provides more evidence for *MEF2B* associated with muscle growth. The SNP was also located within the intron of *RFXANK*, one of the subunits of *RFX*, which is a ubiquitously expressed factor that binds to the promoters of all *MHC* genes. *RFX* helps other transcription factors bind to *MHC* to enhance the binding specificities [48]. *RFXANK* has been proved to highly express in skeletal muscle by SAGE(Serial Analysis of Gene Expression) in human [49].

OAR3_84073899.1 was located within the gene *CAMKMT* (calmodulin-lysine N-methyltransferase), which is a key mediator of calcium-dependent signaling and is subject to regulatory post-translational modifications, including trimethylation of Lys-115[50]. The isoform of *CAMKMT* is expressed in the brain, liver, muscle, colon, and lung, and muscle cells may be regulated by calcium-binding [51].

In our study, OAR3_115712045.1 was identified to within *TRHDE*. *TRHDE* is thyrotropin-releasing hormone degrading enzyme, it encodes a member of the peptidase M1 family. The encoded protein is an extracellular peptidase that specifically cleaves and inactivates the neuropeptide thyrotropin-releasing hormone [46], [52].*TRHDE* was reported to be associated to neuroglioma in human [53]. OAR3_115712045.1 was also located within QTL which has been reported to affect internal fat amount in Merino sheep [54].

OAR9_91721507.1 was located within *RIPK2*, which is receptor-interacting serine-threonine kinase2 that encodes a member of the receptor-interacting protein(RIP) family of serine protein kinases. RIP acts as an activator of nuclear factor kappa B (NF-κB) and a target of activated receptors of the tumor necrosis factor receptor (TNFR) type in myogenic differentiation and regeneration. TNFR associated TRAF6-, IL-1-R-, and TLR-type receptors activate NF-κB. During myogenic differentiation in vitro, *TRAF6* gene expression is down regulated in normal myoblasts, suggesting that *TRAF6* plays a role during this process. Inhibition of *TRAF6* expression using specific siRNAs inhibited both myoblast proliferation and differentiation, whereas inhibition of the *TRAF6* effector NF-κB alone in our system only blocked proliferation. Thus, *PIPK2* may play the same role as *TRAF6* in the regulation of skeletal muscle differentiation and regeneration in sheep [55].Moreover, OAR9_91721507.1 was located within QTL which has been reported to affect hot carcass weight, eye muscle area and muscle weight in carcass in Merino sheep [54].

*GRM1* is a member of the metabotropic class of glutamate receptors and it was found in many species. *GRM1* has been shown to activate phospholipase C (PLC) and is associated with Na^+^ and K^+^ channels. Its action is generally excitatory, causing more glutamate to be released from the presynaptic cell. When PLC is activated by *GRM1*, phosphatidylinositol 4 and 5-bisphosphate in the membrane are hydrolyzed, producing IP3 and 1, 2- sn-diacylglycerol, which plays a role in cell proliferation, early development and differentiation in animals and plants [56].OAR8­_75441328.1 was located with QTL reported to affect internal fat amount in Merino sheep [54].

*POL* gene is one of the structural genes in retroviruses. The genomic region encoding the viral enzymes protease, reverse transcriptase, and integrase. There were reports that *POL* is associated with Ovine Pulmonary Carcinoma(OPC, a respiratory disease in sheep) [57], Maedi-Visna Disease(MVD, a respiratory disease in sheep)[58], [59],Bovine Leukaemia Virus(BLV, a blood disease in bovine), Avian Leukosis (AL, blood disease in chicken) [60], [61]. *POL* gene is the necessary for proliferation of the above disease virus. Once animals infect these virus, the common feature is progressive wasting [62]. Thus we think that *POL* might lead to the change of body weight for a long time before sheep's being affected with some diseases and then influence growth and meat production in sheep indirectly.

*UBR2*: ubiquitin protein ligase E3 component n-recognin 2. This gene encodes an E3 ubiquitin ligase of the N-end rule proteolytic pathway that targets proteins with destabilizing N-terminal residues for polyubiquitylation and proteasome-mediated degradation. No reference was found to tell the relationship between *UBR2* and growth and meat production traits at present.

*MBD5* is Methyl-CpG-binding domain protein 5, it is a member of the methyl-CpG-binding domain (MBD) family. The MBD consists of approximately 70 residues and representing the minimal region required for a methyl-CpG-binding protein to bind methylated DNA. In addition to the MBD domain, this protein contains a PWWP domain (Pro-Trp-Trp-Pro motif), which consists of 100−150 amino acids found in numerous proteins involved in cell division, growth, and differentiation. New research shows that chromosomal abnormalities of MBD5 is associated with autism and schizophrenia [63], [64], [65].

*RPL7* is called ribosomal protein L7. It is a ribosomes that catalyze protein synthesis, this gene encodes a ribosomal protein. The protein belongs to the L30P family of ribosomal proteins and it can inhibit cell-free translation of mRNAs. It is located in the cytoplasm and plays a regulatory role in the translation apparatus. The protein has been shown to be an autoantigen in patients with systemic autoimmune diseases, such as systemic lupus erythematosus. As is typical for genes encoding ribosomal proteins, there are multiple processed pseudogenes of this gene dispersed through the genome. *SMC2* structural maintenance of chromosomes 2, a member of SMC family, is critical for mitotic chromosome condensation in frogs and for DNA repair in mammals [66].

From the above elementary description of the candidate genes, we find some of them are more or less associated with muscle development and body weight in different species, which allows us to predict the genes might take part in similar processes in sheep genome. Generally, similar genes are distributed with the same macroscopic function in different animals or plants in nature, but with differences on the microscopic level. Biologists at New York University have identified how different species use common genes to control their early development and modulate these genes depending on specific cellular requirement [67]. Besides, it seems some other candidate genes have no relationship with the traits we concerned, and we can not find any reference to prove. However, they're just what we want to forecast and focus here. Compared with other species, less genes and functions were researched in sheep, that's why we perform a GWAS to try to explore more novel genes in sheep. To those novel genes, we will attempt to prove their pleiotropism using different ways in next research.

Except the 11 most important candidate genes at genome-wise significance level, other 25 genes (*NLGN1*,*EPB41L3*,*C1ORF87*,*CHMP5*,*LRPPRC, TGIF1*,*STT3A, ADAMTS2, TRPS1*,*SRP68*,*HYDIN, LSM3*,*MYO10*,*CCDC15*,*MSL1*,*NTN1*,*ZWINT, PLA2G6*,*PFKFB4*,*TRDN, OXSM, RARB, LRRC2*,*ADK, SHISA9*)were thought to be the same important genes too. They were identified to be associated with PWG, PRWG, DWG, CG, SC, SMWT, and WWT at chromosome-wise significance level. We think the reasons they did not reach genome-wise significance level are because of the statistical method, sample size, etc. But they are still promising genes for growth and meat production traits.

In addition, it's worth to mention that before the *Ovis_aries*_v3.1 genome sequence released, we used sheep genome *Ovis_aries*_v1.0 to annotate genes(we started the experiment nearly two years ago). Unfortunately, no significant SNPs were found within the ovine genes and the distance from selected SNP to nearest ovine gene (*NR1D1*) is 32kb. Then we referred to the genomic information of other species to forecast the sheep genome. We presumed that the sequences containing the SNPs may code the same genes in sheep by analyzing the gene description and function in human, cattle, mouse, and rat genome. Facts proved that the program was feasible and most of supposed genes in human, bovine, rat and mouse were also found in sheep genome *Ovis_aries*_v3.1 too (Table 4), which will help us to perform deep sequencing of the genes for further investigation.

There were no significant SNPs related to BWT, EMA, FT, or HW. We speculated the following causes: To begin with, so far only 13 QTL were reported for ultrasound fat depth and less QTL for BWT, EMA and HW. Secondly, after quality control, 11% SNPs were excluded, which reduced the density of markers, and 319 individuals is a relatively small sample size in GWAS research. Lastly, SNPs number varies on each chromosome, less significant SNPs were identified on those chromosomes that had less SNPs distribution. Therefore we did not get the meaningful SNPs that related to BWT, EMA, FT, or HW in this study.

Overall, a total of 36 distinct SNPS that were significant at the chromosome-wise level were identified for 7 growth and meat production traits, and 10 of these SNPs reached genome-wise significance level. Among these results, the most valuable outcome may be the 10 SNPs located within five genes *(MEF2B*, *RFXANK*, *CAMKMT*, *TRHDE*, *RIPK2*) and close to six genes (*GRIM1*, *POL*, *MBD5*, *UBR2*, *RPL7* and *SMC2* ).These genes are thought to be the candidate genes which were correlated with sheep growth and meat production traits. This article emphasized the process of the GWAS and the selection of related genes. We just take a preliminary analysis for the candidate genes. The results could be used as a basic foundation to guide follow-up replication studies. Subsequent studies including exploring, analysis of network, and functional verification will be done in the candidate genes, which could ultimately reveal the causal mutations underlying meat-production traits in sheep.
